# Type 2 Nep1-Like Proteins from the Biocontrol Oomycete *Pythium oligandrum* Suppress *Phytophthora capsici* Infection in Solanaceous Plants

**DOI:** 10.3390/jof7070496

**Published:** 2021-06-22

**Authors:** Kun Yang, Xiaohua Dong, Jialu Li, Yi Wang, Yang Cheng, Ying Zhai, Xiaobo Li, Lihui Wei, Maofeng Jing, Daolong Dou

**Affiliations:** 1 The Key Laboratory of Plant Immunity, College of Plant Protection, Nanjing Agricultural University, Nanjing 210095, China; 2019202016@njau.edu.cn (K.Y.); 2019202017@njau.edu.cn (X.D.); 2019102008@njau.edu.cn (J.L.); 2020102017@stu.njau.edu.cn (Y.W.); cy1562513941@163.com (Y.C.); 2 Department of Plant Pathology, Washington State University, Pullman, WA 99164, USA; ying.zhai@wsu.edu; 3 Crops Research Institute, Guangdong Academy of Agricultural Sciences/Guangdong Provincial Key Laboratory of Crop Genetic Improvement, Guangzhou 510640, China; lixiaobo1981@163.com; 4 Institute of Plant Protection, Jiangsu Academy of Agricultural Sciences, Nanjing 210014, China; weilihui@jaas.ac.cn

**Keywords:** Nep1-like proteins, *Pythium oligandrum*, *Phytophthora capsici*, solanaceous plants, oomycete resistance

## Abstract

As a non-pathogenic oomycete, the biocontrol agent *Pythium oligandrum* is able to control plant diseases through direct mycoparasite activity and boosting plant immune responses. Several *P. oligandrum* elicitors have been found to activate plant immunity as microbe-associated molecular patterns (MAMPs). Necrosis- and ethylene-inducing peptide 1 (Nep1)-like proteins (NLPs) are a group of MAMPs widely distributed in eukaryotic and prokaryotic plant pathogens. However, little is known about their distribution and functions in *P. oligandrum* and its sister species *Pythium periplocum*. Here, we identified a total of 25 NLPs from *P. oligandrum* (PyolNLPs) and *P. periplocum* (PypeNLPs). Meanwhile, we found that PyolNLPs/PypeNLPs genes cluster in two chromosomal segments, and our analysis suggests that they expand by duplication and share a common origin totally different from that of pathogenic oomycetes. Nine PyolNLPs/PypeNLPs induced necrosis in *Nicotiana* *benthamiana* by agroinfiltration. Eight partially purified PyolNLPs/PypeNLPs were tested for their potential biocontrol activity. PyolNLP5 and PyolNLP7 showed necrosis-inducing activity in *N. benthamiana* via direct protein infiltration. At sufficient concentrations, they both significantly reduced disease severity and suppressed the in planta growth of *Phytophthora capsici* in solanaceous plants including *N. benthamiana* (tobacco), *Solanum lycopersicum* (tomato) and *Capsicum annuum* (pepper). Our assays suggest that the *Phytophthora* suppression effect of PyolNLP5 and PyolNLP7 is irrelevant to reactive oxygen species (ROS) accumulation. Instead, they induce the expression of antimicrobial plant defensin genes, and the induction depends on their conserved nlp24-like peptide pattern. This work demonstrates the biocontrol role of two *P. oligandrum* NLPs for solanaceous plants, which uncovers a novel approach of utilizing NLPs to develop bioactive formulae for oomycete pathogen control with no ROS-caused injury to plants.

## 1. Introduction

Oomycetes are a large and diverse group of Stramenopiles, which display fungal-like morphology. Most oomycetes are devastating pathogens of plants or animals [1,2,3]. For example, *Phytophthora capsici* causes root, crown, foliar and fruit rot on important vegetables such as pepper (*Capsicum annuum*), tomato (*Solanum lycopersicum*), pumpkin and cucumber. Oomycete disease incidence and severity have increased significantly in recent decades and caused substantial losses in agriculture throughout the world [4]. Interestingly, two non-pathogenic oomycetes, *Pythium oligandrum* and *Pythium periplocum*, are effective mycoparasites [5,6,7,8]. *P. oligandrum* colonizes the root ecosystem of diverse plants to induce defense responses [6]. It also produces antimicrobial compounds, competes for nutrients and space with pathogens, and promotes plant growth via producing the auxin precursor tryptamine [6,9,10]. Thus, *P. oligandrum* has been successfully used in agriculture for plant disease control [6,11,12].

Similar to pathogenic oomycetes, *P. oligandrum* rapidly infects plant root tissues. However, its subsequent degeneration does not cause host tissue damage. As a result, *P. oligandrum* protects plants from various biotic stresses via inducing local and systemic resistance [6,13,14]. One way that plants obtain induced resistance is mediated by pathogen- or microbe-associated molecular patterns (PAMPs or MAMPs), which are delivered from pathogenic or non-pathogenic microbes into the extracellular matrix of the plant cell. As the first layer of immunity, plants utilize a large set of pattern recognition receptors (PRRs) at the cell surface to perceive PAMPs or MAMPs, and activate PAMP- or MAMP-triggered immunity (PTI or MTI), respectively [15]. To date, only two types of MAMPs, oligandrins and the *P. oligandrum* D-type of cell wall protein fractions (CWPs)(POD-1 and POD-2), are identified and characterized in *P. oligandrum*. They both belong to the elicitin-like family found in *Phytophthora* and *Pythium* species [16,17,18,19]. However, *P. oligandrum* oligandrins and *POD-1/2* are species-specific and distinct from elicitin or elicitin-like proteins in pathogenic oomycetes [18]. None of their encoding genes, including two CWP genes (*POD-1/2*) and two oligandrin genes (*Oli-D1* and *Oli-D2*), are present in the genomes of known pathogenic *Pythium* species [6,20]. Both oligandrins and *POD-1/2* can induce hypersensitive cell death and reactive oxygen species (ROS) accumulation in hosts, which enhance plant resistance against several pathogens including *Phytophthora nicotianae*, *Fusarium oxysporum* and *Botrytis* *cinerea* [21,22,23].

In addition to oligandrins and *POD-1/2*, there are other MAMP candidates in non-pathogenic oomycetes. Necrosis and ethylene-inducing peptide 1 (Nep1)-like proteins (NLPs) form a superfamily of microbial proteins produced by taxonomically diverse organisms, including bacteria, fungi, and oomycetes [24,25,26]. Multiple NLPs are known to behave as virulence factors and MAMPs to activate immune responses in dicotyledons [26,27]. NLPs feature an N-terminal signal peptide that directs proteins to the secretory pathway for delivery into the extracellular environment, and a C-terminal conserved NPP1 domain (PF05630) with a conserved heptapeptide motif (GHRHDWE) in its central region [26]. NLPs have cytotoxic and noncytotoxic forms. The cytotoxic NLPs bind glycosyl inositol phosphoryl ceramide (GIPC) sphingolipids as cytolytic, to induce necrosis in eudicots but not in monocots. The function of noncytotoxic NLPs remains poorly understood [26,28]. The cytotoxic NLPs in certain hemibiotrophic plant pathogens such as *P. capsici* and *Verticillium dahliae* are considered to be essential for their full virulence and the transition to necrotrophic stages during infection [29,30]. Plants such as *Arabidopsis thaliana* have evolved to recognize the conserved NLP peptides (nlp20 and nlp24) of NLPs as MAMPs to trigger immunity responses including mitogen-activated protein kinase (MAPK) activation and ROS burst [31,32]. For example, an *A. thaliana* leucine-rich repeat receptor-like protein (LRR-RLP) RLP23 can recognize nlp20, which is conserved in cytotoxic and noncytotoxic NLPs from bacteria, fungi, and oomycetes [31]. However, neither an nlp20 response nor an RLP23 ortholog can be identified in solanaceous plants (*Nicotiana benthamiana*, tomato, potato, etc.), wheat, or *Arabidopsis lyrate*, the sister species of *A. thaliana* [26,32], suggesting that nlp20/RLP23-mediated immune activation is not conserved across plant species. Interestingly, RLP23 can recognize nlp20-containing NLPs in heterologous plant systems. The stable expression of *A. thaliana* RLP23 in potato is able to significantly reduce the growth of *Phytophthora infestans*, which encodes a number of NLPs carrying nlp20 [31].

Oomycete genomes have an expanded set of *NLPs* due to extensive gene duplications. For example, the soybean root rot pathogen *Phytophthora sojae* contains 70 *NLP* genes with multiple duplicated copies clustered in the genome [30]. In contrast, the rice blast fungus *Magnaporthe oryzae* encodes merely 4 *NLPs* [24,33,34], and only 1 to 2 *NLPs* can be found in most bacteria [26]. NLPs can be divided into three distinct types based on the occurrence of conserved cysteine residue pairs in the N-terminal [24,25]. Type 1, 2 and 3 NLPs have one, two and three pairs of conserved cysteines, respectively [24,35]. Most oomycete species, such as the plant pathogens *P. sojae*, *Pythium ultimum* and *Pythium aphanidermatum*, encode type 1 NLPs. As the most abundant NLP type, type 1 NLPs also occur in bacteria and fungi [25,26]. Type 2 NLPs were originally thought to be only present in bacteria and fungi, but absent in oomycetes. However, recent analyses demonstrate their occurrence in *P. oligandrum* [1,35]. Phylogenetic analyses of amino acid sequences suggest a diverse evolution of NLPs from *P. oligandrum* and other oomycetes [26,35]. It is still unknown whether NLPs act as MAMPs in nonpathogenic *P. oligandrum* and its sister species *P. periplocum*.

Here, we identified and cloned type 2 NLP-encoding genes in *P. oligandrum* strain CBS 530.74 (*PyolNLP1~14*) and *P. periplocum* strain CBS 532.74 (*PypeNLP1~11*) based on the analysis of their sequenced genomes [36,37]. Necrosis-inducing activity was seen in 9 out of 25 *PyolNLPs*/*PypeNLP*s in *N. benthamiana*. Furthermore, we explored the biocontrol roles of recombinant necrotic PyolNLP proteins and found that PyolNLP5 and PyolNLP7 were able to suppress the in planta infection of *P. capsici*. Our work uncovers a novel type (NLP) of MAMP proteins from biocontrol agents without the side effect of ROS injury. The newly identified NLPs can be used to develop bioactive formulae for disease control practice in solanaceous plants.

## 2. Materials and Methods

### 2.1. DNA Cloning, Plasmid Construction and Peptide Synthesis

The full-length cDNA sequences of all *PyolNLPs* and *PypeNLPs* were amplified from *P. oligandrum* strain CBS 530.74 and *P. periplocum* strain CBS 532.74 by polymerase chain reaction (PCR). Fragments used to generate PyolNLP-M24 mutants were synthesized by Nanjing Genscript (Nanjing, China). Amplified fragments were cloned into pBINHA (a plasmid vector containing an HA tag under the control of the Cauliflower mosaic virus 35S promoter) using an In-Fusion^®^ HD Cloning Kit (Clontech, Mountain View, CA, USA). The coding regions of *NLPs* and *NLP* mutants without the signal peptide were amplified and inserted into *Escherichia coli* expression vector pET32a to fuse with a His-tag. Individual colonies for each construct were tested for insertions by PCR. Selected clones were verified by DNA sequencing. Primers used in this work were listed in Table S3.

### 2.2. Bioinformatics Analysis

The NLP sequences were aligned using the muscle method of MEGA6 with the default parameters first. The aligned sequences were then subjected to MEGA6 to construct the phylogenetic tree following the neighbor-joining algorithm, with 1000 bootstrap replicates. The protein sequences of PyolNLPs/PypeNLPs were submitted to SignalPv3.0 (http://www.cbs.dtu.dk/services/SignalP/) (accessed on 06 August 2019) for secreted signal peptide prediction. The conserved nlp24 sequence, the heptapeptide motif, and key residues responsible for necrosis activity were identified, based on previous reports [24,38].

### 2.3. SDS-PAGE and Western Blots

Proteins from the sample lysate were fractionated by sodium dodecyl sulfate–polyacrylamide gel electrophoresis (SDS-PAGE). Fractionated proteins were electro-transferred from the gel to an Immobilon-PSQ polyvinylidene difluoride membrane using transfer buffer (20 mM Tris, 150 mM glycine). The membrane was then blocked for 30 min at room temperature using phosphate-buffered saline (PBS; pH 7.4) containing 3% nonfat dry milk with shaking at 50 rpm. After one wash with PBST (PBS with 0.1% Tween 20), an anti-HA (1:2000, Abmart) antibody was added to PBSTM (PBS with 0.1% Tween 20 and 3% nonfat dry milk) and incubated for 90 min. After three washes (5 min each) with PBST, the membrane was then incubated with goat anti-mouse IRDye 800CW antibodies (Odyssey) at a ratio of 1:10,000 in PBSTM for 30 min. The membrane was finally washed with PBST and visualized with excitations at 700 and 800 nm.

### 2.4. Purification and Quantification of Recombinant PyolNLP Proteins

Recombinant constructs were transformed into *E. coli* strain Rosetta-gamiB. Expression of 6xHis-tagged PyolNLPs and mutant proteins was induced by adding 0.1 mM isopropyl-β-d-thiogalactopyranoside (IPTG) and incubating at 37 °C for 6 h. Soluble His-tagged proteins were purified by affinity chromatography using Ni-NTA Agarose (Qiagen) according to the manufacturer’s instructions. Protein standard bovine serum albumin (BSA) solution with a range of 0.5 μg/μL to 2.0 μg/μL was used to make a standard curve, to compare protein samples. Partially purified proteins (5 μL) and the standard BSA solution in PBS buffer were separated by SDS-PAGE. Gels were stained with coomassie solution (1 g of coomassie brilliant blue R250, 250 mL of isopropanol, 100 mL of acetic acid, and 650 mL of deionized water) for 2 h with gentle shaking, and then destained in solution (10 % (*v/v*) of acetic acid and 5 % (*v/v*) of ethanol) with gentle shaking. Transparent SDS-PAGE gels with clear bands were photographed under natural light. Next, we quantify the concentration of protein in SDS-PAGE gel bands using ImageJ software (http://rsb.info.nih.gov/ij/) (accessed on 21 September 2017) as described previously [39].

### 2.5. Agrobacterium Tumefaciens Infiltration Assays in Nicotiana benthamiana

Constructs were transformed into *Agrobacterium tumefaciens* strain GV3101 by electroporation. Successful transformants were confirmed by PCR amplification using indicated primers (Table S1). Transformed *A. tumefaciens* strains were cultured, washed, and re-suspended in infiltration buffer (10 mM MgCl_2_, 500 mM MES, 100 mM acetosyringone) to make an appropriate optical density (OD) of 0.3 at 600 nm. *N. benthamiana* plants were infiltrated with a 1:1 mixture of resuspended *A. tumefaciens* containing the respective constructs and a suppressor of the gene silencing (P19) strain. In this study, *N. benthamiana* plants were grown and maintained in growth chambers with an ambient temperature of 23 °C under a 16-h light/8-h dark photoperiod. Infiltration assays were conducted on 4-week-old *N. benthamiana* leaves using a needleless syringe. To prepare plant samples for gene expression analysis, agro-infiltrated leaf samples were collected at given time intervals and immediately frozen with liquid nitrogen before being stored for further study.

### 2.6. Oomycete Inoculation Assay

For *P. oligandrum* inoculation in *N. benthamiana*, mycelia plugs were incubated in 10% (*v/v*) V8 juice medium at 25 °C for 2 days and inoculated into *N. benthamiana* leaves under 25 °C in the dark for 3 to 72 hpi. For the pathogenicity assay in *N. benthamiana*, detached leaves from 4-week-old *N. benthamiana* plants were inoculated with mycelia plugs of *P. capsici* isolate LT263 and *P. nicotianae* isolate 025, and then incubated under controlled environmental conditions (25 °C; dark). Inoculated leaves were photographed under bright or UV light at 36 or 48 hpi. Lesion diameters were measured with the ImageJ software. All experiments were repeated with three independent biological replicates, with at least 12 leaves per replicate, respectively. For a pathogenicity assay in the fruits of the Hang pepper and cherry tomato, partially purified proteins of the same concentration (6 μM) were sprayed evenly on the fruit surface. Fruits were then inoculated with equal amounts of *P. capsici* mycelium plugs. The inoculated fruits were kept in plastic boxes with a relative humidity of 90~100% for 48 h to 72 h. The boxes were placed at 25 °C in the dark. Inoculated pepper fruits were photographed at 48 and 60 hpi, and the lesion lengths were measured at the indicated time points. At 72 hpi, lesions were cut longitudinally to obtain their depth grades. For infected tomatoes, disease severity readings were taken at 48 or 60 hpi, by visual inspection and measuring the percentage of the damaged area. The disease index was calculated according to the following formula: DI = [(Σ disease score × amount of infected fruit)/total checked leaves × 9] × 100 [40]. The symptoms were evaluated, and the disease score was classified as follows: 0, no symptoms; 1, lesion area < 5%; 3, lesion area 5% to 10%; 5, lesion area 10% to 20%; 7, lesion area 20% to 50%; 9, lesion area > 50%. All experiments were repeated with three independent biological replicates, with at least 15 fruits per replicate, respectively.

### 2.7. DAB Staining and Electrolyte Leakage Assay

For DAB staining, *N. benthamiana* leaves were stained with 1 mg/mL DAB solution for 8 h in the dark at 12 hpi and then decolored with ethanol for light microscopy examination. Samples were equilibrated with 70% (*v/v*) glycerol for photography using natural light. For electrolyte leakage assay, five leaf discs (9 mm in diameter) of *N. benthamiana* were soaked in 5 mL of distilled water for 2 h at room temperature. The conductivity of the bathing solution was then measured using a conductivity meter (Con 700; Consort, Tutnhout, Belgium).

### 2.8. RNA Isolation and qRT-PCR

Total RNA samples were extracted from *P. oligandrum* and *N. benthamiana* leaves by using the RNA-simple Total RNA Kit (Tiangen) according to the manufacturer’s instructions. cDNA was synthesized using the HiScript 1st Strand cDNA Synthesis Kit (Vazyme). Real-time PCR was performed by using the ChamQ SYBR qPCR Master Mix kit (Vazyme) and the ABI Prism 7500 Fast real-time PCR system, following the manufacturer’s instructions. The gene-specific primers used for qRT-PCR and their purposes are listed in Table S3.

### 2.9. Statistical Analysis of Data

SPSS 22 software was used for statistical analysis of all data. The results were analyzed by a median-edition Levene’s test to determine the homogeneity of variances across groups, and then analyzed by one-way ANOVA with a post hoc Tukey’s range test for groups with equal variances, or Kruskal–Wallis test analysis for groups with unequal variance (*, *p* < 0.05; **, *p* < 0.01; ns, no significant differences). The results are the means ± s.d. of replicates.

## 3. Results

### 3.1. The Occurrence of Type 2 NLPs in Pythium oligandrum and Pythium periplocum

**Figure 1:**
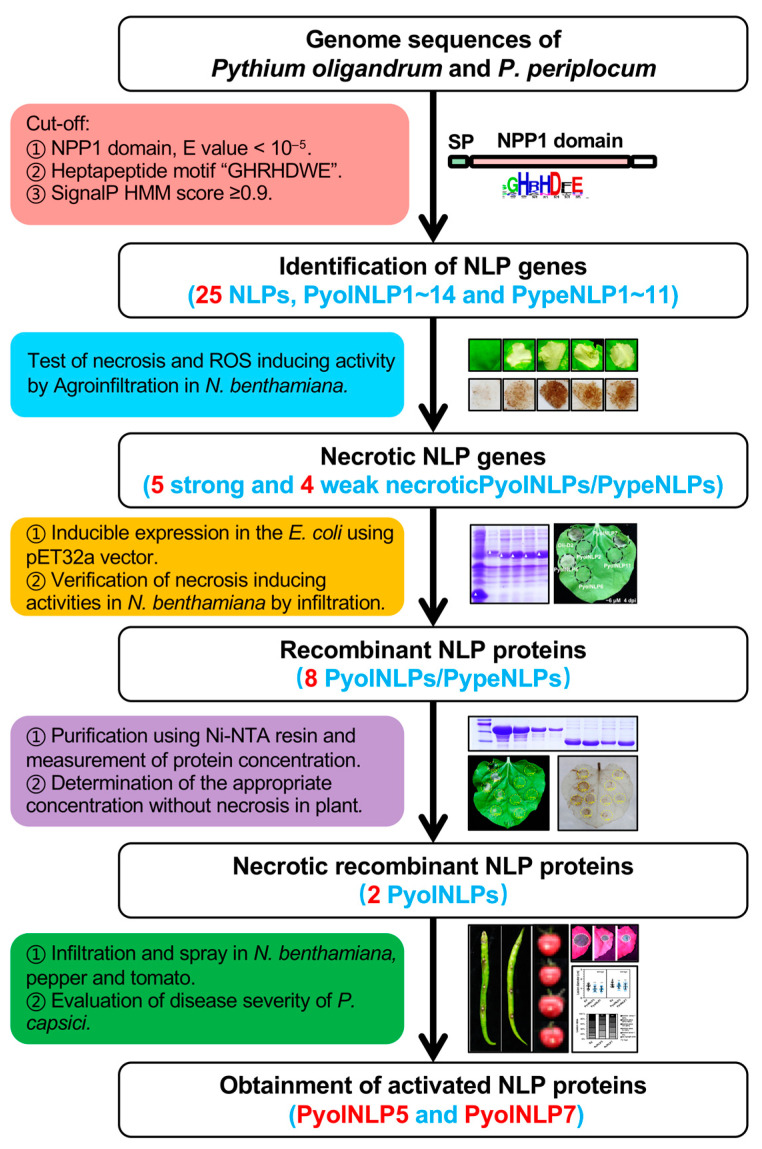
Strategy for identifying bioactive Nep1-like proteins (NLPs) from *Pythium oligandrum* and *Pythium periplocum*. Candidate NLP genes were identified from *P. oligandrum* and *P. periplocum* genomes using a bioinformatics pipeline. They were then agroinfiltrated in *N. benthamiana* leaves to screen necrosis-inducing NLPs. Necrotic NLPs screened out were expressed in *E. coli* and purified for functional assays, including direct infiltrations in *N. benthamiana* for the determination of their threshold concentrations for necrosis induction and disease suppression evaluation. Finally, the bioactive NLP proteins selected were used to infiltrate and spray solanaceous plants. Disease severity was evaluated after *P. capsici* inoculation.

**Figure 2:**
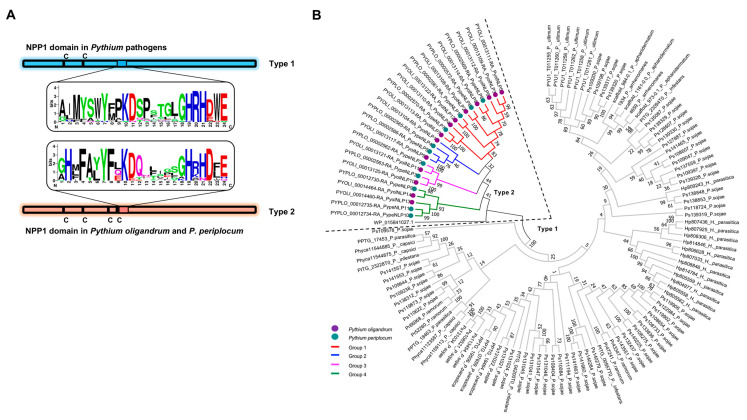
All predicted Nep1-like proteins (NLPs) in *Pythium oligandrum* and *Pythium periplocum* belong to type 2. (**A**) Schematic representation of the type 1 and type 2 NPP1 domains from *Pythium* pathogens and nonpathogenic *P. oligandrum* and *P. periplocum*. The number and position of conserved cysteine residues were shown. The graphic representation of conserved region nlp24 within the NPP1 domain contains the conserved heptapeptide motif ‘‘GHRHDWE’’. The web-logo (http://weblogo.berkeley.edu/) is based on 21 type 1 NLP sequences from *Pythium* pathogens (including *P. ultimum*, *P. aphanidermatum*, *P. arrhenomanes* and *P. irregulare*) and 25 type 2 NLP sequences from *P. oligandrum* and *P. periplocum*. “C” indicates the conserved cysteine residues. Amino acids are represented as single-colored letters. Letter height corresponds to its appearance frequency at a particular position. (**B**) The phylogenetic tree shows the diversification of NLPs among oomycetes. The association of individual sequences to taxonomic groups of NLPs from *P. oligandrum* and *P. periplocum* is indicated by colors. The two different NLP types are labeled. The NLP sequences are from *H. parasitica*, *P. sojae*, *P. capsici*, *P. nicotianae*, *P. infestans*, *P. ramorum*, *P. ultimum*, *P. aphanidermatum*, and *P. arrhenomanes*.

For the identification of NLPs in *P. oligandrum* and *P. periplocum*, the hidden Markov model of the NPP1 domain (PFAM: PF05630) was obtained from the PFAM database, and used to search the proteomes of *P. oligandrum* strain CBS 530.74 and *P. periplocum* strain CBS 532.74 [36,37] with a cut-off Expect (*E*) value of 10^−5^. Meanwhile, the SignalP v3.0 program was used to characterize N-terminal signal peptides with a cut-off value of 0.90 (Figure 1). In total, 16 and 11 NPP1-containing proteins were identified in *P. oligandrum* and *P. periplocum*, respectively (Figure 1). Among them, 14 and 11 proteins were predicted to contain a signal peptide and were named PyolNLP1~14 and PypeNLP1~11, respectively (Table S1, Figure 1). In contrast, pathogenic *Pythium* species contain roughly four NLPs in general [26,35]. As shown in Figure 2A, all PyolNLPs/PypeNLPs contained two pairs of conserved cysteine residues in their NPP1 domains, suggesting the exclusive occurrence of type 2 NLPs in these two biocontrol *Pythium* agents. In contrast, only type 1 NLPs occur in pathogenic *Pythium* species, including *Pythium ultimum*, *Pythium aphanidermatum*, *Pythium arrhenomanes* and *Pythium irregulare* [26,35]. Taken together, pathogenic and non-pathogenic *Pythium* species have evolved two distinct types of NLPs with dramatically different copy numbers. Both type 1 and type 2 NLPs have a conserved peptide pattern, nlp24 [24]. All *Pythium* NLPs examined contain the nlp24 peptide of 24 to 27 amino acids in length, which include a conserved “GHRHDF/LE” motif. A unique “GHxFAYYFxKDQ” motif occurs in the N-terminal regions of PyolNLPs/PypeNLPs, whereas pathogenic *Pythium* NLPs contain an “AIMYSWYFPKDSP” motif typically found in other type 1 and type 2 NLPs [38] (Figure 2A).

The amino acid sequences of 25 PyolNLPs/PypeNLPs were used to construct a neighbor-joining phylogenetic tree, together with a type 2 NLP in *Pectobacterium carotovorum* [24] and 100 representative NLPs from 9 oomycete pathogens, including *Hyaloperonospora parasitica*, *P. sojae*, *P. capsici*, *P. nicotianae*, *P. infestans*, *Phytophthora ramorum*, *P. ultimum*, *P. aphanidermatum* and *P. arrhenomanes* [26,35]. Phylogenetic analysis clearly distinguishes the diversification of oomycete NLPs. All 25 PyolNLPs/PypeNLPs are clustered together with the type 2 NLP of *P. carotovorum* while all type 1 NLPs from pathogenic oomycetes form another clade (Figure 2B). In addition, we found that PyolNLPs/PypeNLPs can be divided into four groups, with high homology exhibited within each group (Figure 2B).

**Figure 3:**
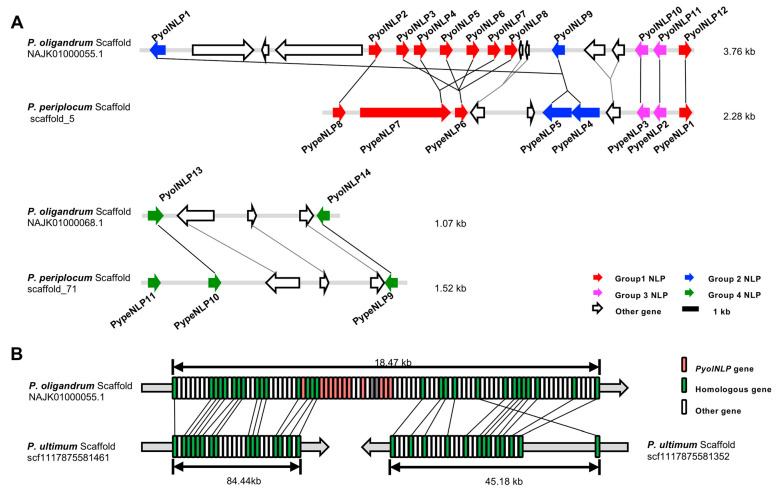
Synteny of *PyolNLPs/PypeNLPs* genes. (**A**) The *PyolNLPs/PypeNLPs* genes occur in clusters in the genomes of *P. oligandrum* and *P. periplocum*, showing the number and position of conserved cysteine residues. Colored arrows indicate the *NLP* genes with different groups. Lines join syntenic genes with the same orientation. Staggered black lines show the scaffold joins predicted from the synteny analysis. (**B**) The analysis of collinearity of the flanking sequence of the NLP clustered region in *P. oligandrum* and *P. ultimum*. The region around *PyolNLP1~12* spans *P. oligandrum* scaffold NAJK01000055.1 and *P. ultimum* scaffolds scf1117875581461 and scf1117875581352. Lines join syntenic genes with the same orientation.

*Phytophthora NLP* genes tend to be clustered in the genome. For example, 36 out of 70 predicted *NLPs* in *P. sojae* occurred in groups of two or more with the rest 34 *NLPs* being more randomly distributed around the genome as singletons [30]. In contrast, all *PyolNLP*/*PypeNLP* genes were found to cluster in two chromosomal segments (Figure 3A, Supplementary Table S1). *PyolNLP1~12* is localized in scaffold NAJK01000055.1 of *P. oligandrum*. Its homolog *PypeNLP1~8* is localized in scaffold_5 of *P. periplocum*. Similarly, *PyolNLP13~14* and its homolog *PypeNLP9~11* are localized in scaffold NAJK01000068.1 and scaffold_71, respectively (Figure 3A, Supplementary Table S1). In pathogenic oomycetes such as *P. sojae*, elicitin genes are located adjacent to *PsNLP* clusters [30]. Due to the non-pathogenic nature of *P. oligandrum* and *P. periplocum*, there is no effector or elicitin gene families co-localized with *NLP* genes in their genomes (Figure 3A). Interestingly, most *PyolNLP*/*PypeNLP* genes in the same group clustered closely (Figure 2B and Figure 3A).

It is conceivable that the occurrence of type 2 NLPs in *P. oligandrum* is the consequence of an additional, independent horizontal gene transfer (HGT), likely from bacteria, not the other *Pythium* or *Phytophthora* pathogens [26,35]. To gain more insight, we blasted the genome of *P. ultimum*, the sister species of *P. oligandrum*, which only contains six NLPs. As shown in Figure 3B, we analyzed the collinearity of the flanking sequence of the NLP clustered region in *P. oligandrum*. Both the 5′ and 3′ flanking sequences showed high collinearity with the pathogenic *P. ultimum* (Figure 3B, Table S2). In contrast, no conserved synteny could be found in the NLP clustered region. These results further support the hypothesis that the NLP genes of *P. oligandrum* and *P. periplocum* did not originate from oomycete species.

### 3.2. Nine PyolNLPs/PypeNLPs Show Necrosis-Inducing Activity in Nicotiana benthamiana

**Figure 4:**
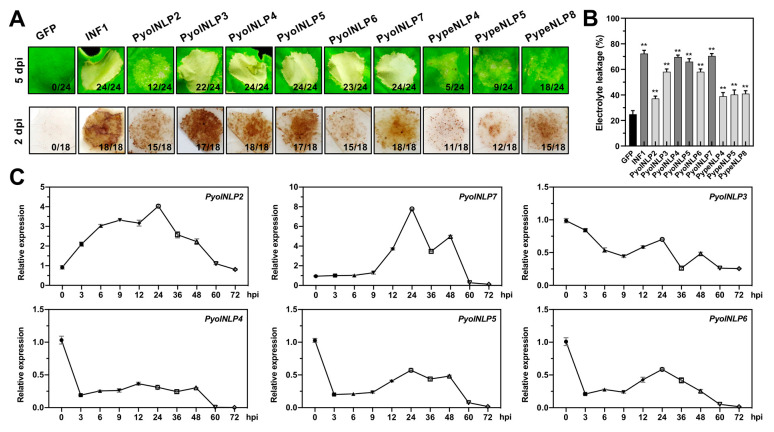
The PyolNLPs and PypeNLPs from Groups 1 and 2 show cytotoxic activity in *Nicotiana benthamiana* as revealed by transient expression assays. (**A**) The necrosis-inducing activity (upper panel) and H_2_O_2_ accumulation (lower panel) of PyolNLPs/PypeNLPs. Ratios indicate the proportion of infiltrated sites that developed the cell death phenotype. The number of days post-infiltration (dpi) with different recombinant *A. tumefaciens* that contain the NLP genes is indicated. Results from the expression of a negative control of green fluorescent protein (GFP) and a positive control of *P. infestans* PAMP INF1 are also shown for comparison. All experiments were repeated 18 or more times. (**B**) Quantification of electrolyte leakage in *N. benthamiana* leaves expressing cytotoxic PyolNLPs/PypeNLPs (5 days after agroinfiltration). Electrolyte leakage from the infiltrated leaf discs was measured as a percentage of leakage from boiled discs. Error bars represent the mean ± s.d. (*n* = 3). The data were analyzed by a median-edition Levene’s test to determine the homogeneity of variances across groups, and then analyzed by one-way ANOVA with a post hoc Tukey’s range test for groups with equal variances (**, *p* < 0.01). Experiments were repeated three times with similar results. (**C**) Expression profile of *PyolNLPs* during *P. oligandrum* inoculation of leaves. Total RNA was extracted from mycelia (MY) or inoculated *N. benthamiana* leaves at 3, 6, 9, 12, 24, 36, 48, 60 and 72 h post-inoculation (hpi). Transcript levels of *PyolNLPs* were determined by qRT-PCR. The *P. oligandrum Actin* gene was used as the reference.

Cytolytic NLPs share a common feature of activating immune responses [26]. To determine the cytotoxic activities of PyolNLPs/PypeNLPs, their open reading frames (ORFs) were fused with an HA tag in pBINHA for *A. tumefaciens*-mediated transient expression in *N. benthamiana* (Figure 1). *P. infestans* PAMP INF1 and GFP were used as positive and negative controls, respectively. Nine PyolNLPs/PypeNLPs induced necrosis in *N. benthamiana* leaves, with five of them causing strong symptoms (Figure 4A and Supplementary Figure S1). Consistent results were obtained when monitoring necrosis induction activity using ion leakage (Figure 1 and Figure 4B). A 3,3′-diaminobenzidine (DAB) staining assay on leaves infiltrated with these nine necrosis-inducing PyolNLPs/PypeNLPs detected significant H_2_O_2_ accumulation at 48 h post-infiltration (hpi) (Figure 4A). Western blots showed that all NLPs were correctly expressed in *N. benthamiana* (Supplementary Figure S1). Interestingly, all nine necrotic PyolNLPs/PypeNLPs fall in Groups 1 and 2, suggesting their common origins and expansion via duplication (Figure 2B and Figure 3A). Quantitative reverse transcription PCR (qRT-PCR) assays on six cytotoxic *PyolNLPs* revealed their distinct expression patterns during *P. oligandrum*-*N. benthamiana* interactions (0 to 72 hpi) (Figure 4C). *PyolNLP2* and *PyolNLP7* were upregulated upon inoculation, with peak expression around 24 hpi. In contrast, *PyolNLP3~6* was downregulated during the interaction.

### 3.3. Recombinant PyolNLP5 and PyolNLP7 Proteins Suppress Phytophthora Pathogen Infection in N. benthamiana Leaves

**Figure 5:**
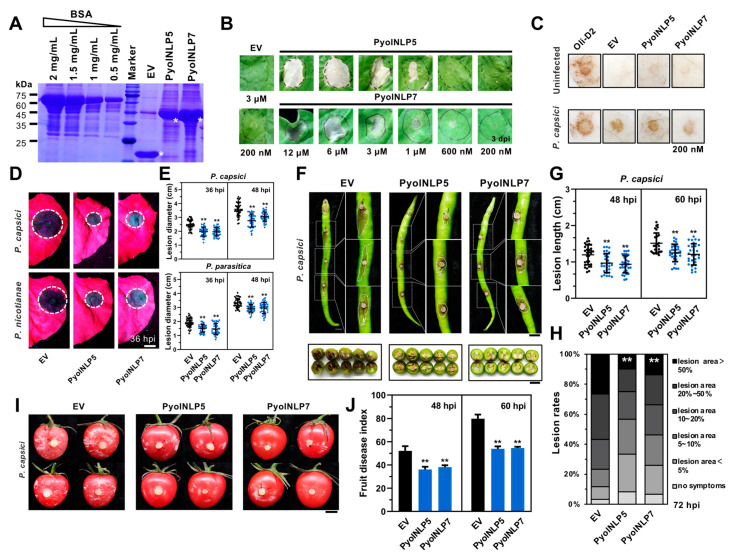
Recombinant PyolNLP5 and PyolNLP7 proteins suppress the infection of *Phytophthora* pathogens in *Nicotiana benthamiana*, pepper and tomato fruits. (**A**) SDS-PAGE analysis of PyolNLP5 and PyolNLP7 proteins stained with coomassie blue. Recombinant proteins were expressed in *E. coli* using the pET32a vector and partially purified using Ni-NTA resin. Asterisks indicate the objective bands. (**B**) Representative *N. benthamiana* leaves infiltrated with partially purified PyolNLP5 or PyolNLP7 proteins (200 nM to 12 μM). Partially purified protein from an empty vector (EV) with His-tag was used as a negative control. Photographs were taken at 3 dpi. (**C**) H_2_O_2_ accumulation on *N. benthamiana* leaves infiltrated with PyolNLP5 or PyolNLP7. *P. capsici* was inoculated at 0 or 12 hpi. Partially purified His-tag protein acts as a negative control. Oli-D2 acts as a positive control. DAB staining was performed at 12 hpi. (**D**) Phenotypes of *N. benthamiana* leaves infiltrated with PyolNLP5 or PyolNLP7 and followed by the inoculation of *P. capsici* or *P. nicotianae*. Photos were taken at 36 hpi. Partially purified His-tag protein was used as a negative control for infiltration. (**E**) Lesion diameters were measured by ImageJ. Data were analyzed from 40 biological repeats. (**F**) Top: Phenotypes of pepper fruits sprayed with 6 μM PyolNLP5 or PyolNLP7 and followed by *P. capsici* inoculation. Representative photos were taken at 60 hpi. Bottom: transection shows the disease severity of pepper fruit rot caused by *P. capsici*. (**G**) Lesions of pepper fruits inoculated with *P. capsici* at 48 and 60 hpi. (**H**) The fruit rot ratings of pepper fruit rot caused by *P. capsici*. The disease symptoms were recorded at 72 dpi. Disease ratings are shown in different colors. (**I**) Phenotypes of tomato fruits sprayed with PyolNLP5 or PyolNLP7 followed by *P. capsici* inoculation. Representative photos were taken at 60 hpi. (**J**) The fruit disease index of tomato fruit rot caused by *P. capsici*. The disease symptoms were recorded at 60 dpi. Bar = 1 cm. Error bars represent the mean ± s.d. The data were analyzed by a median-edition Levene’s test to determine the homogeneity of variances across groups, and then analyzed by one-way ANOVA with post hoc Tukey’s range test for groups with equal variances, or Kruskal–Wallis test analysis for groups with unequal variance; *, *p* < 0.05; **, *p* < 0.01. All experiments were repeated at least three times.

We then tested the necrosis-inducing ability of partially purified PyolNLPs/PypeNLPs by directly infiltrating protein solution (Figure 1). Eight out of nine PyolNLPs/PypeNLPs that induce necrosis in *N. benthamiana* by agroinfiltration could be successfully expressed and partially purified from *Escherichia coli* using the pET32a vector (Table 1 and Supplementary Figure S2A). Among them, only partially purified PyolNLP5 and PyolNLP7 were able to induce cell death in *N. benthamiana* by infiltration with about 6 μM recombinant proteins at 4 dpi (Table 1 and Supplementary Figure S2B). Gradient assays showed that the cell death-inducing ability of recombinant PyolNLP5 and PyolNLP7 proteins (Figure 5A) were positively correlated with protein concentrations, with both of them failing to trigger necrosis or ROS burst at 200 nM (Figure 5B,C).

To test the *Phytophthora* pathogen suppression ability of PyolNLP5 and PyolNLP7, *N. benthamiana* leaves were pre-treated with 200 nM PyolNLP5 or PyolNLP7 (Figure 5D) before inoculating with the *P. capsici* strain LT263 at 12 hpi. Compared to the control of the partially purified His-tag, *N. benthamiana* leaves infiltrated with either PyolNLP5 or PyolNLP7 exhibited smaller lesions (Figure 5D,E) at both 36 and 48 hpi, suggesting that both PyolNLPs are able to reduce disease severity and suppress the in planta growth of *P. capsici*. Meanwhile, neither PyolNLP5 nor PyolNLP7 could induce ROS burst upon *P. capsici* infection (Figure 5C). PyolNLP5 and PyolNLP7 can also suppress the colonization of *P. nicotianae* strain 025 in *N. benthamiana* (Figure 5D,E).

### 3.4. Recombinant PyolNLP5 and PyolNLP7 Proteins Suppress Phytophthora capsici Infection in Pepper and Tomato Fruits

Under favorable environmental conditions, *P. capsici* can produce a large number of sporangia to infect the fruit surface and cause fruit rot. Solanaceae plants such as pepper and tomato are especially vulnerable to *P. capsici*-induced fruit rot [4]. Here, we assessed the fruit rot controlling activity of PyolNLP5 and PyolNLP7 using Hang pepper and cherry tomato, both of which are popular vegetables in China. Compared to the negative control, pepper fruits sprayed with partially purified PyolNLP5 or PyolNLP7 showed decreased disease lesions, lower disease indexes, and attenuated water-soaking phenotypes at 48 and 60 hpi after inoculation of *P. capsici* (Figure 5F–H). *P. capsici* also showed decreased pathogenicity on cherry tomato fruits sprayed with partially purified PyolNLP5 or PyolNLP7 at 48 and 60 hpi (Figure 5I,J). Our results indicate that PyolNLP5 and PyolNLP7 suppress *P. capsici* infection and reduce disease severity on the fruits of Solanaceae plants.

### 3.5. PyolNLP5 and PyolNLP7 Suppress Phytophthora Pathogen Infection via Inducing Plant Defensin Expression in an nlp24-Dependent Manner

**Figure 6:**
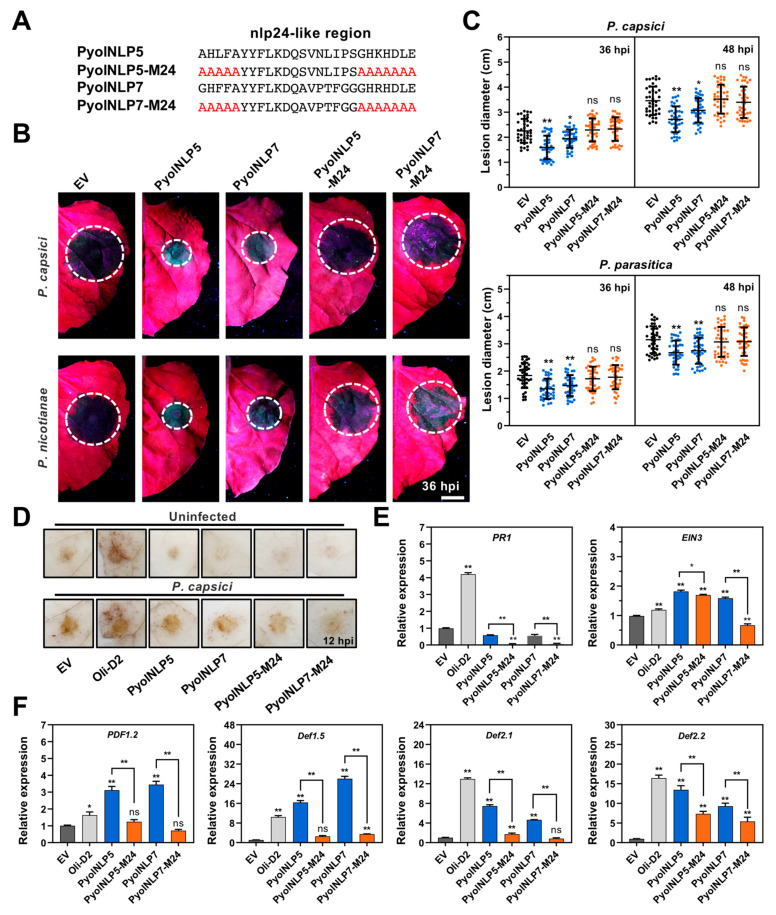
PyolNLP5 and PyolNLP7 suppress *Phytophthora* pathogen infection in *Nicotiana benthamiana* leaves via inducing plant defensin expression in an nlp24-dependent manner. (**A**) Schematic representation of nlp24 in PyolNLP5 and PyolNLP7 with introduced alanine substitutions. (**B**) Phenotypes of *N. benthamiana* leaves infiltrated with 200 nM PyolNLP5, PyolNLP7 or their nlp24 mutant proteins, and followed by *P. capsici* or *P.* *nicotianae* inoculation. Photos were taken at 36 hpi. Partially purified protein from EV with His-tag was used as a negative control. (**C**) Lesion diameters were measured by ImageJ. Data were analyzed from 40 biological repeats. (**D**) H_2_O_2_ accumulation on *N. benthamiana* leaves infiltrated with partially purified PyolNLP5/7 or PyolNLP5/7-M24 mutant proteins. *P. capsici* was inoculated at 0 or 12 hpi. Oli-D2 acts as a positive control. DAB staining was performed at 12 hpi. (**E**,**F**) Relative expression levels of *PR1*, *EIN3* and plant defensins genes in *N. benthamiana* leaves infiltrated with partially purified PyolNLP5, PyolNLP7 or their mutant proteins. Partially purified His-tag protein acts as a negative control. Partially purified Oli-D2 acts as a positive control. Bar = 1 cm. Error bars represent the mean ± s.d. The results were analyzed by Median-edition Levene’s test to determine the homogeneity of variances across groups, and then analyzed by one-way ANOVA with a post hoc Tukey’s range test for groups with equal variances; *, *p* < 0.05; **, *p* < 0.01; ns, no significant differences. Experiments were repeated three times with similar results.

Despite the fact that *N. benthamiana* does not respond to nlp24-triggered immunity [32], the conserved nlp24 pattern plays an important role in the function of NLPs, with its first four amino acids (AIMY) and the GHRHDWE motif both being essential for NLP activity [32,38,41,42]. Here, we mutated the conserved sites of the nlp24-like peptide pattern in PyolNLP5 and PyolNLP7 to create PyolNLP5-M24 and PyolNLP7-M24, respectively (Figure 6A). Both Pyolnlp24-mutated NLPs were expressed in *E. coli*, partially purified using the Ni-NTA resin, and then infiltrated into *N. benthamiana* leaves at a concentration of 200 nM (Supplementary Figure S3). As compared to the negative control, both PyolNLP5-M24 and PyolNLP7-M24 failed to enhance plant resistance against *P. capsici* or *P.* *nicotianae* (Figure 6B,C).

Oligandrins (Oli-D1 and Oli-D2) from *P. oligandrum* can activate plant defense responses including ROS burst and the upregulation of defense responsive genes, such as *pathogenesis-related protein 1* (*PR1*) and *lipoxygenase* (*LOX*) [21,22,23]. In contrast, neither PyolNLP5/7 nor their M24 mutants stimulated H_2_O_2_ accumulation in infiltrated plants as compared to the negative control (Figure 6D). The infection of *P. capsici* showed consistent results (Figure 6D). These results suggest that PyolNLP5/7 enhances plant resistance irrespective of the ROS burst.

We further examined transcript accumulation changes of key plant defense responses upon *P. capsici* infection, including *Cyp71D20* and *PTI5* as involved in PTI [43] (Supplementary Figure S4A), *Enhanced disease susceptibility 1* (*EDS1*) and *PR1* involved in salicylic acid signaling pathway [44] (Supplementary Figure S4B, Figure 6E), and *Ethylene Insensitive 3 (EIN3)*, *plant defensin 1.2* (*PDF1.2*), *LOX*, *PR2*, *PR3* and *PR4* involved in jasmonic acid and ethylene signaling pathways [45] (Supplementary Figure S4C, Figure 6E,F). Only *PDF1.2* and its upstream regulatory gene *EIN3* [45] exhibited significant upregulation in PyolNLP5/7-infiltrated plants (Figure 6E,F). PyolNLP5/7-induced upregulation of *PDF1.2* and *EIN3* can be attenuated/abolished by the M24 mutation (Figure 6E,F).

**Figure 7:**
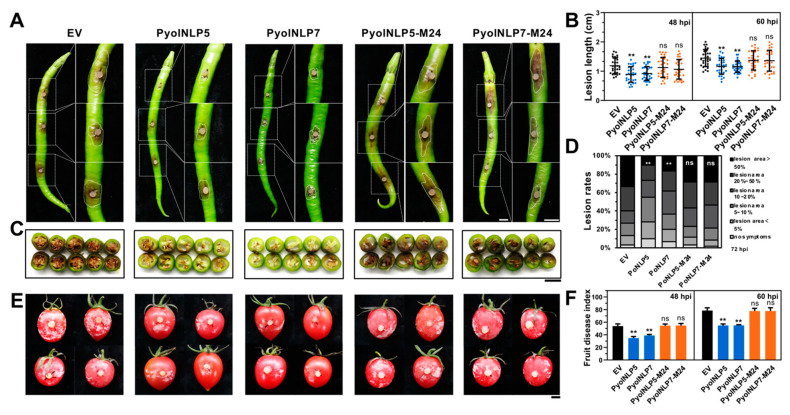
The nlp24 region is required for PyolNLP5- and PyolNLP7-mediated suppression of *Phytophthora* pathogen infection in pepper and tomato fruits. (**A**) Phenotypes of pepper fruits sprayed with 6 μM PyolNLP5, PyolNLP7, PyolNLP5-M24 or PyolNLP7-M24 proteins and followed by *P. capsici* inoculation. Representative photos were taken at 60 hpi. (**B**) Lesions of pepper fruits inoculated with *P. capsici* at 48 and 60 hpi. Data were analyzed from 40 biological repeats. (**C**) Transection shows the disease severity of pepper fruit rot caused by *P. capsici*. (**D**) Ratings of pepper fruit rot caused by *P. capsici*. The disease symptoms were recorded at 72 dpi. Disease ratings are shown in different colors. (**E**) Phenotypes of tomato fruits sprayed with 6 μM PyolNLP5, PyolNLP7 or their mutant proteins, and followed by *P. capsici* inoculation. Representative photos were taken at 60 hpi. (**F**) The fruit disease index of tomato fruit rot caused by *P. capsici*. The disease symptoms were recorded at 60 dpi. Bar = 1 cm. Data were analyzed from 40 biological repeats. Error bars represent the mean ± s.d. The data were analyzed by a median-edition Levene’s test to determine the homogeneity of variances across groups, and then analyzed by one-way ANOVA with a post hoc Tukey’s range test for groups with equal variances, or Kruskal–Wallis test analysis for groups with unequal variance; **, *p* < 0.01; ns, no significant differences. Experiments were repeated three times with similar results.

The expression of three *N. benthamiana defensin* genes, *NbDef1.5*, *NbDef2.1* and *NbDef2.2*, was significantly induced by either PyolNLP5 or PyolNLP7 (Figure 6F). Similarly, the induction can be impaired by the M24 mutation (Figure 6F). As major components of the plant’s innate immune system [46,47], defensins are small, basic and cysteine-rich proteins with direct antimicrobial activities. *P. capsici* infection can induce the expression of several *N. benthamiana defensin* genes [48]. Our results suggest that PyolNLP5 and PyolNLP7 suppress *Phytophthora* infection via inducing plant defensin expression, which is dependent on their nlp24 region. Furthermore, PyolNLP5-M24 and PyolNLP7-M24 failed to enhance *P. capsici* resistance in pepper and tomato fruits (Figure 7). The result indicated that the Solanaceae fruit protection effect of PyolNLP5 and PyolNLP7 is also nlp24-dependent.

## 4. Discussion

*P. oligandrum* and its sister species *P. periplocum* are soilborne free-living oomycetes capable of parasitizing pathogenic fungi and oomycetes [6,7,49]. *P. oligandrum* can also colonize endophytically the root tissues of diverse plants to induce defense responses via MAMPs [6,50]. Both species are used as biocontrol agents for fungal and oomycete disease management in Solanaceae crops. In this research, we identify NLP-encoding genes (*PyolNLPs*/*PypeNLPs*) from these two non-pathogenic oomycetes, and reveal their underlying gene duplication events and an evolutionary theme distinct from that of previously defined NLPs in pathogenic *Pythium* and *Phytophthora* species. Nine PyolNLPs/PypeNLPs are characterized as novel species-specific MAMPs, in addition to previously described oligandrins and *POD-1/2* [6,18,20]. In particular, partially purified PyolNLP5 and PyolNLP7 proteins are capable of reducing disease severity and suppressing in planta growth of *Phytophthora* pathogens in solanaceous plants. We further reveal that their downstream acting mechanism is irrelevant to ROS burst but via inducing plant defensin gene expression. Our findings provide a non-ROS injury and transgene-free approach for *Phytophthora* pathogen control in solanaceous plants.

The first NLP identified is from the vascular wilt fungus *F. oxysporum* that induces ethylene biosynthesis and necrosis in plants [51]. NLP-family members widely occur in various bacteria, fungi, and oomycete microbes. They have an extremely broad taxonomic distribution, even within the oomycete species. Type 1 NLPs are found exclusively in phytopathogenic oomycetes, whereas all NLPs in nonpathogenic *P. oligandrum* and *P. periplocum* belong to type 2, and are similar to a cytotoxic NLP homolog from the bacterium *P. carotovorum*. Hence, type 2 *NLP* genes in *P. oligandrum* are proposed to be horizontally transferred from bacteria [26,35], which is supported by the results of our phylogenetic analysis. Oomycete *NLP* genes undergo striking expansion, with most species harboring more than 10 *NLPs* [26]. For example, the majority of 70 *NLPs* found in *P. sojae* are derived from recent duplication events occurring in closely proximal chromosomal segments [30]. Meanwhile, we found that both *PyolNLPs* and *PypeNLPs* are highly homologous within the group and closely clustered in two chromosomal segments of their respective genomes. Furthermore, most *PyolNLP*/*PypeNLP* genes clustered in the same group exhibit similar necrosis-inducing activity, suggesting their common origins are derived from extensive gene duplications. Consistent with the non-pathogenic nature of *P. oligandrum* and *P. periplocum*, their *NLPs* do not colocalize with effector or elicitin genes like their counterparts in pathogenic oomycetes, related gene families such as RXLR effectors, CRN effectors or elicitins within *NLP* genes in genomes. Our gene organization and evolutionary analysis results suggest that *PyolNLPs* and *PypeNLPs* genes expand by duplication and share a common origin totally different from that of pathogenic oomycetes. Gene duplications lead to genetic redundancy and thereby facilitate the emergence of novel or altered functions [26]. Certain PyolNLPs/PypeNLPs may have developed their MAMP, necrosis induction and *Phytophthora* pathogen suppression functions via this approach.

The expansion and rapid diversion of NLPs also suggest their important roles in the oomycete infection process. Cytotoxic NLP-induced necrosis could be beneficial for hemibiotrophic *Phytophthora* pathogens, which thrive on dead plant tissues at their necrotrophic infection stages [26]. Cytolytic *NLPs* are found to express at the biotrophic-to-necrotrophic switch stages during infection. For example, cytolytic *PiNPP1.1* in *P. infestans* is upregulated during the late stages of tomato infection [52]. *P. sojae NLPs* are also highly expressed during late infection stages [30]. Most cytolytic *PcNLPs* are highly expressed when necrotic lesions occur in pepper leaves infected by *P. capsici*. On the other hand, the silencing of *PcNLPs* impairs *P. capsici* virulence in pepper [53]. These results lead to the hypothesis that cytolytic NLPs function as phytotoxins in the host necrosis induction and the transition to necrotrophic stages during infection [26]. NLPs also contribute to virulence in various pathogens other than oomycetes. For example, *NLP* knockout mutants of the bacterial pathogen *P. carotovorum* exhibit reduced virulence [42]. Cytolytic NLPs from the vascular wilt pathogen *V. dahliae* are notable for their full virulence on the tomato, but not on cotton [29,54]. The Nep1-like protein family of *M. oryzae* is notable for its virulence on rice [34].

For the nonpathogenic *P. oligandrum*, two cytolytic *PyolNLPs* (*PyolNLP2* and *PyolNLP7*) are upregulated during the infection of *N. benthamiana* leaves until 48 hpi. Their subsequent downregulation at 60 and 72 hpi is coincident with the cellulose degradation of *P. oligandrum* [55]. Unlike most cytolytic *NLPs* in pathogenic oomycetes whose expression is elevated at necrotrophic stages [30,53], four cytolytic *PyolNLPs* are significantly downregulated upon infection. All *Pythium* oomycetes can rapidly infect plant root tissues, but only pathogenic species (e.g., *P. aphanidermatum*) are able to kill host tissue within hours. The repression of cytolytic NLPs in non-pathogenic *Pythium* species might fail to induce host tissue damage during the infection process.

In the present study, agroinfiltration of 9 PyolNLPs/PypeNLPs showed necrotic activity in *N. benthamiana*, to further confirm the activity of these PyolNLPs/PypeNLPs, and to minimize potential influences on protein production by the *A. tumefaciens*-mediated expression system, we examined the ability of in vitro-purified recombinant proteins to induce necrosis in *N. benthamiana*. The results showed that only two partially purified proteins (i.e., PyolNLP5 and PyolNLP7) produced in *E. coli* were able to induce cell death in *N. benthamiana*. MAMPs trigger broad resistance to diverse pathogens [15], for example, ethylene-inducing xylanase (EIX)-like protein 3 from the soilborne fungus *V. dahliae* (VdEIX3) can induce cell death and ROS burst, and increase resistance against oomycetes and fungal pathogens in *N. benthamiana* [56]. The elicitin-like proteins oligandrins and *POD-1/2* from *P. oligandrum* can induce cell death and ROS accumulation, which enhance plant resistance against several oomycetes and fungal pathogens [21,22,23]. Although the exogenous application of partially purified PyolNLP5 and PyolNLP7 proteins at a non-ROS injury concentration (200 nM) onto plants did not induce any visible cell death or ROS accumulation, they revealed efficacies in reducing disease severity caused by *P. capsici* in solanaceous plants, including *N. benthamiana*, tomato and pepper. This should be an advantage for the development of bioactive formulae for disease control, because significant necrosis or ROS injury caused by bioactive proteins may not be acceptable for practical use. Further tests also revealed that PyolNLP5 and PyolNLP7 proteins were effective in suppressing *P.* *nicotianae* infection in *N. benthamiana*. Together, the results suggest that PyolNLP5 and PyolNLP7 have a promising future for controlling *P. capsici* and *P.* *nicotianae*. However, further studies are needed to evaluate the efficacies of PyolNLP5 and PyolNLP7 in suppressing fungal pathogens.

Our gene expression analysis reveals that PyolNLP5/7 modulates plant defense via inducing the expression of *defensins* and *EIN3*, the upstream regulator of *PDF1.2*. Plant defensins are toxic to pathogens but not mammalian or plant cells [46,47]. Defensins render resistance to a broad range of pathogens via their diverse antifungal, antibacterial, α-amylase and trypsin inhibitory activities [57]. For example, plant defensins interact with the fungal membrane to induce ion leakage, Ca^2+^ signaling, MAPK activation, ROS production, and ultimately the death of fungal cells [57]. Defensins have been widely employed as an effective strategy for disease control in plants. Several antifungal defensins have been adopted for the development of transgenic crops resistant to phytopathogens including a few *Phytophthora* species [58]. For example, papaya plants expressing *Dahlia merckii* defensin DmAMP1 exhibit improved resistance to *Phytophthora palmivora* by reducing pathogen vigor [59]. Transgenic tobacco expressing mustard defensin BjD is resistant to *P.* *nicotianae* [60]. In this work, we show that PyolNLP5 and PyolNLP7 are able to significantly induce the expression of multiple plant *defensins* including *PDF1.2*, *NbDef1.5*, *NbDef2.1* and *NbDef2.2*, but not genes involved in other typical defense pathways, suggesting that PyolNLP-mediated defense regulation is largely specific to defensins.

Our mutation analysis demonstrates that the key amino acid sequence of Pyolnlp24 is essential for the plant resistance enhancement function of PyolNLP5 and PyolNLP7. In *A. thaliana*, plant RLP23 can recognize the conserved nlp24 peptide pattern of NLPs to trigger plant immunity responses, including MAPK activation, ROS burst and defense gene expression [31,32]. However, this recognition mode is not conserved across plant species. In particular, no RLP23 ortholog can be found in *N. benthamiana*. In addition, ROS burst is not one of the downstream responses induced by PyolNLP5/7. Thus, nlp24 may play a novel role in the recognition of PyolNLP5/7 by their partner(s). One possible scenario is that PyolNLPs can be recognized by unknown receptor(s) in *N. benthamiana* and other solanaceous plants, and then enhance *Phytophthora* resistance in a non-classical defensin-dependent manner. PyolNLP-receptor interactions are likely to be mediated by the conserved nlp24 pattern. The detailed mechanism of the interactions is yet to be determined.

## 5. Conclusions

In conclusion, our work represents the first comprehensive research on NLPs of biocontrol oomycetes. The phylogenetics of NLPs and their roles in necrosis induction and disease suppression have been investigated extensively. Among them, PyolNLP5 and PyolNLP7 are able to reduce oomycete infection in solanaceous plants, even in the form of isolated proteins. Their acting mechanisms are irrelevant to the ROS burst but are closely related to defensins, which makes them ideal candidates for developing novel biocontrol agents.

## Figures and Tables

**Table 1:** Summary of the necrosis-inducing activity of partially purified NLP proteins in *Nicotiana benthamiana*.

ID	Predicted Molecular Weight (kDa)	Necrosis-Inducing Activity by Agroinfiltration	Inducible Expression in *Escherichia coli*	Necrosis-Inducing Activity by Infiltration of Recombinant Protein
PyolNLP2	45.9	Yes	Yes	No
PyolNLP3	45.8	Yes	Yes	No
PyolNLP4	45.1	Yes	Yes	No
PyolNLP5	45.7	Yes	Yes	Yes
PyolNLP6	45.9	Yes	Yes	No
PyolNLP7	45.4	Yes	Yes	Yes
PypeNLP4	46.4	Yes	Yes	No
PypeNLP5	46.5	No	No	No
PypeNLP8	45.9	Yes	Yes	No

## Data Availability

The data presented in this study are available on request from the corresponding author.

## Supplementary Materials

The following are available online at https://www.mdpi.com/article/10.3390/jof7070496/s1. Figure S1: Screening of the necrotic PyolNLPs/PypeNLPs. Figure S2: Recombinant PyolNLP5 and PyolNLP7 induce necrosis in *N. benthamiana*. Figure S3: SDS-PAGE analysis of PyolNLP5-24 and PyolNLP7-M24 proteins stained with coomassie blue. Figure S4: Relative expression levels of key genes related to plant defense in *N. benthamiana* leaves infiltrated with partially purified PyolNLP5, PyolNLP7 or their mutant proteins. Table S1: Summary of 14 and 11 necrosis- and ethylene-inducing-like protein (NLP) genes in the genomes of *Pythium oligandrum* and *Pythium periplocum*, respectively. Table S2: Collinearity analysis of the left and right arms of NLP clustered region. Table S3: Primers used in this study.

## Abbreviations

MAMPs	Microbe-associated molecular patterns
NLPs	Necrosis- and ethylene-inducing peptide 1 (Nep1)-like proteins
ROS	Reactive oxygen species
PAMPs	Pathogen-associated molecular patterns
PRRs	Pattern recognition receptors
PTI	PAMP-triggered immunity
MTI	MAMP-triggered immunity
CWPs	Cell wall protein fractions
POD	*P. oligandrum* D-type of CWPs
GIPC	Glycosyl inositol phosphoryl ceramide
LRR-RLP	Leucine-rich repeat receptor-like protein
ORF	Open reading frame
PCR	Polymerase chain reaction
qRT-PCR	Quantitative reverse transcription PCR
SDS-PAGE	Sodium dodecyl sulfate polyacrylamide gel electrophoresis
PBS	Phosphate-buffered saline
IPTG	Isopropyl-β-d-thiogalactopyranoside
BSA	Bovine Serum Albumin
OD	Optical density
HGT	Horizontal gene transfer
DAB	3,3′-diaminobenzidine
GFP	Green fluorescent protein
hpi	hours post-inoculation
dpi	days post-inoculation
MY	mycelia
EV	Empty vector
PR1	Pathogenesis-related protein 1
LOX	Lipoxygenase
EDS1	ENHANCED DISEASE SUSCEPTIBILITY 1
EIN3	ETHYLENE INSENSITIVE 3
PDF1.2	Plant defensin 1.2
EIX	Ethylene-inducing xylanase
