# Supplementary material for: Type 2 Nep1-Like Proteins from the Biocontrol Oomycete Pythium oligandrum Suppress Phytophthora capsici Infection in Solanaceous Plants
Source: J Fungi (Basel). 2021 Jun 22;7(7):496. doi: 10.3390/jof7070496 (PMC8303654; doi:10.3390/jof7070496)
Supplement: Supplementary file 1 [file jof-07-00496-s001.zip › Supplementary figures.pdf]

Supplementary Information

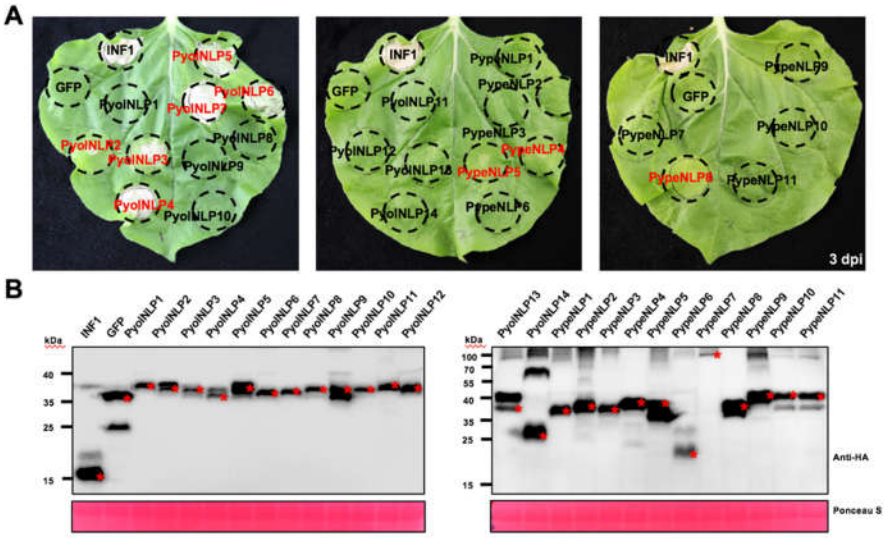

Supplementary Figure S1. Screening of the necrotic PyolNLPs/PypeNLPs. **(A)** *Agrobacterium tumefaciens* infiltration assay for screening PyolNLPs/PypeNLPs inducing necrosis. *PyolNLPs/PypeNLPs*, the cell death-inducing gene *INF1*, and the empty control (GFP) were expressed in *N. benthamiana* leaves via agroinfiltration. **(B)** The expression of all *PyolNLPs/PypeNLPs* in *N. benthamiana* leaves were was confirmed by Western blotting using anti-HA antibody. * indicates objective bands.

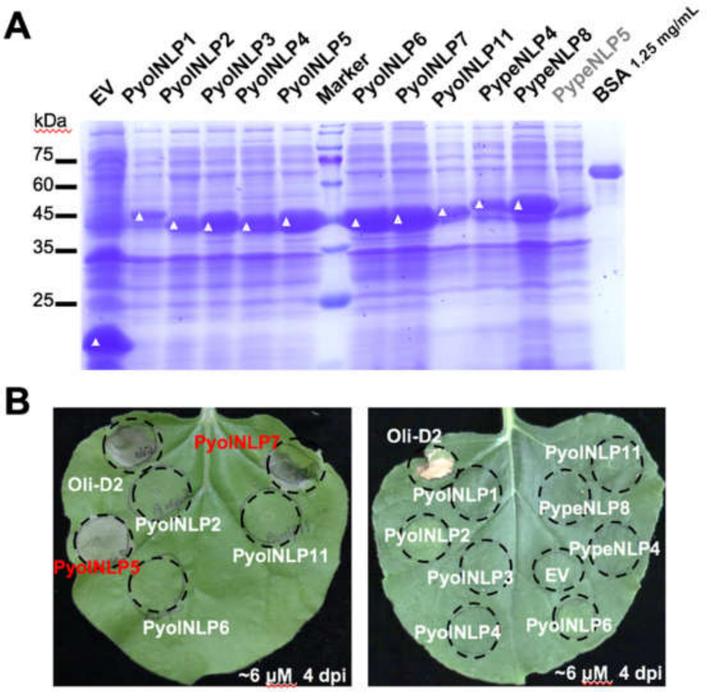

Supplementary Figure S2. Recombinant PyolNLP5 and PyolNLP7 induce necrosis in *N. benthamiana*. **(A)** SDS-PAGE analysis of NLP proteins stained with Coomassie blue. Recombinant proteins were produced in the *Escherichia coli* using the pET32a vector. Asterisks indicate the objective bands. **(B)** Representative *N. benthamiana* leaves infiltrated with about 6 μM purified proteins. Photographs were taken at 4 dpi.

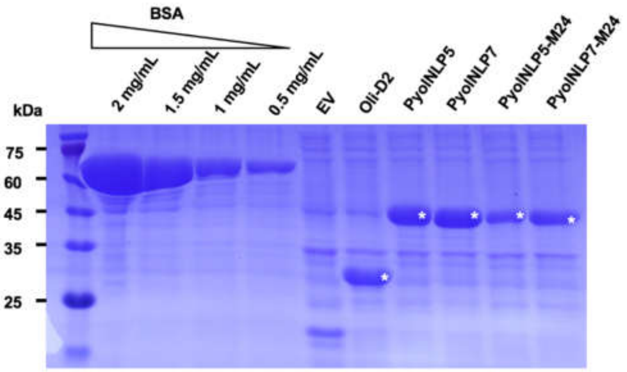

Supplementary Figure S3. SDS-PAGE analysis of PyolNLP5-24 and PyolNLP7-M24 proteins stained with Coomassie blue. Recombinant proteins were expressed in the *Escherichia coli* using the pET32a vector and purified using Ni-NTA resin.

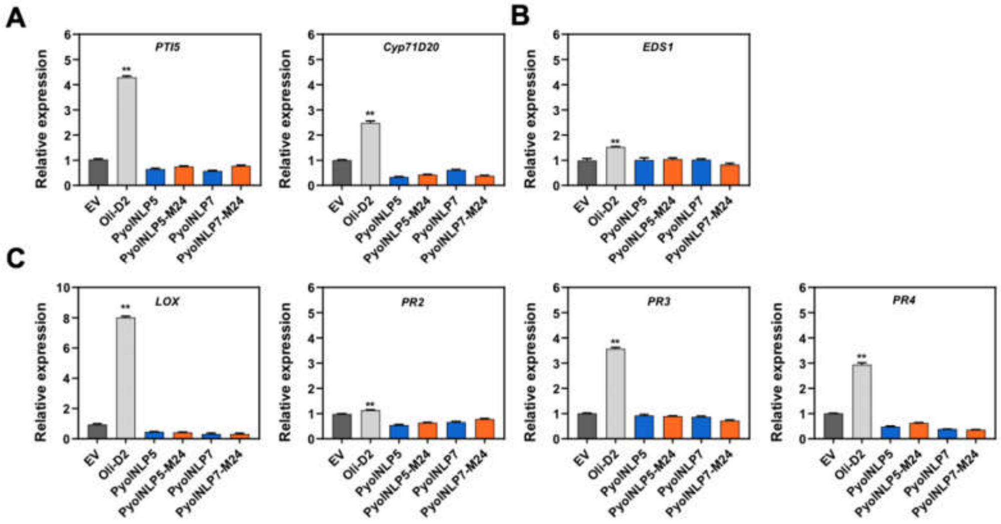

Supplementary Figure S4. Relative expression levels of key genes related to plant defense in *N. benthamiana* leaves infiltrated with partially purified PyolNLP5, PyolNLP7 or their mutant proteins. **(A)** Relative expression levels of PTI-involved *Cyp71D20* and *PTI5*. **(B)** Relative expression level of *EDS1*, which is involved in salicylic acid pathway. **(C)** Relative expression levels of *LOX*, *PR2*, *PR3*, *PR4*, which are involved in jasmonic acid and ethylene pathways. Purified His tag protein acts as a negative control. Purified Oli-D2 acts as a positive control. Leave samples were collected at 12 hpi. Transcription levels were determined by RT-qPCR using *EF1α* as the reference. Error bars represent the mean ± s.d. (n = 3). The results were analyzed by Median-edition Levene's test to determine the homogeneity of variances across groups, and then analyzed by one-way ANOVA with post-hoc Tukey's range test for groups with equal variances; **, P<0.01. Experiments were repeated three times with similar results.

Supplementary Table S1. Summary of 14 and 11 necrosis- and ethylene-inducing-like protein (NLP) genes in the genomes of *Pythium oligandrum* and *Pythium periplocum*, respectively.

Supplementary Table S2. Collinearity analysis of the left and right arms of NLP clustered region (Related to Figure 3B).

Supplementary Table S3. Primers used in this study.
